# Risk Factors Associated with Diabetes among Mexican-Origin Adults in Southern Arizona

**DOI:** 10.3390/ijerph20126126

**Published:** 2023-06-14

**Authors:** Mario Morales, Maia Ingram, Ramses Sepulveda, Thomas Nuño, Ada M. Wilkinson-Lee, Jill E. Guernsey De Zapien, Scott Carvajal

**Affiliations:** 1 Arizona Prevention Research Center, Health Promotion Sciences Department, Mel & Enid Zuckerman College of Public Health, The University of Arizona, Tucson, AZ 85724, USAtnuno@email.arizona.edu (T.N.); adaw@arizona.edu (A.M.W.-L.); dezapien@arizona.edu (J.E.G.D.Z.); carvajal@arizona.edu (S.C.); 2 Department of Mexican American Studies, The University of Arizona, Tucson, AZ 85724, USA

**Keywords:** diabetes, Mexican-origin adults, social determinants of health, US–Mexico border

## Abstract

Diabetes is the seventh leading cause of death in the United States, and it is particularly problematic among the Latine population. This study employed multivariable logistic regression models to examine how hypertension, depression, and sociodemographics were associated with diabetes in a cross-sectional sample of Mexican-origin adults living in three counties of Southern Arizona. The overall prevalence of diabetes from this primary care sample was 39.4%. Holding covariates at fixed values, individuals having hypertension were 2.36 (95% CI: 1.15, 4.83) times more likely to have diabetes, when compared to individuals not having hypertension. The odds of having diabetes for individuals with ≥12 years of educational attainment were 0.29 (95% CI: 0.14, 0.61) times the corresponding odds of individuals with <12 years of educational attainment. For individuals with depression, the odds of having diabetes for those who were born in Mexico and had <30 years living in the US were 0.04 (95% CI: 0, 0.42) times the corresponding odds of individuals without depression and who were born in the US. Findings suggest clinical and public health systems should be aware of the potential increased risk of diabetes among Mexican-origin adults with hypertension and lower educational attainment.

## 1. Introduction

Diabetes is a chronic disease characterized by elevated levels of blood glucose that damage the heart, blood vessels, eyes, kidneys, and nerves [1]. Worldwide, approximately 422 million people have diabetes, and 1.5 million people die each year due to this disease [1]. Diabetes is the seventh leading cause of death in the United States (US) population, and it is particularly harmful among the Latine population [2,3,4]. Between 2017 and 2018, the prevalence of diagnosed diabetes was higher among Latine adults (12.5%) than among non-Latine whites (7.5%) [5], which contributed to the former’s premature mortality and lower quality of life [4]. To the best of our knowledge, the only U.S./Mexico border-wide diabetes prevalence study was carried out at the beginning of the twenty-first century and found a prevalence of 15.7% [6]. A decade ago, the prevalence of diabetes among two cohorts of Mexican-origin adults living in the US–Mexico border, Lower Rio Valley Grande, Texas, and Douglas, Arizona, was between 21% and 31%, respectively [2,4]. Given the limited literature on US–Mexico border studies on diabetes, this paper examines the prevalence of and risk factors associated with diabetes in three counties of Southern Arizona.

Diabetes and hypertension are risk factors for cardiovascular diseases [7], and cardiovascular diseases are the leading cause of death globally [8]. Nowadays, the prevalence of diagnosed hypertension is 47.3% among US adults [9], and the prevalence of hypertension in US adults with diabetes is 66% to 76.3% [10]. Between 2001 and 2002, the prevalence of hypertension along the US–Mexico border was 21.8%, and almost half of the adults with diabetes had hypertension [7]. However, there is a need to update this information. Additionally, diabetes and hypertension are the most prevalent risk factors for stroke among Mexican-origin adults in the US–Mexico border [11,12,13]. Compared to their counterparts, Mexican-origin adults with diabetes and hypertension were more likely to have strokes and ischemic electrocardiographic abnormalities in Corpus Christi and Brownsville, Texas, respectively [11,13]. These findings show that the study of comorbid diabetes and hypertension is important to prevent other diseases, such as stroke among Mexican-origin adults.

Individuals with diabetes are vulnerable to the development of depression and anxiety on a global scale [14,15,16,17]. The prevalence of depression among diabetic patients worldwide ranges from 19% to 22% [18], while the pooled prevalence of anxiety disorders among this population is estimated to be 28% (95% CI: 26, 31, n = 57) [19]. Among the Latine population in the US, particularly among Mexican-origin adults, the comorbidity of diabetes and depression is prevalent [20,21,22]. In three district cohorts in Texas (n = 2436), the prevalence of comorbid depressive symptoms and diabetes was found to be 5 to 12 times higher among elderly Mexican-origin individuals compared to non-Latine whites [23]. Moreover, Mexican-origin individuals with comorbid diabetes and depression were 2.72 (95% CI: 1.08, 7) to 8.42 (95% CI: 2.97, 23.88) times more likely to receive a diagnosis of mild cognitive impairment and 5.14 (95% CI: 1.05, 25.17) to 10.25 (95% CI: 3.14, 33.5) times more likely to develop Alzheimer’s disease when compared to their counterparts [23]. Additionally, a study conducted among a cohort of Mexican-origin adults residing in the Southwest (Texas, Arizona, California, Colorado, and New Mexico, n = 2823) demonstrated that those with diabetes lasting for ≥21 years and experiencing moderate/severe depression exhibited the most significant increase in activities of daily living (ADL) disability [24]. These findings emphasize the importance of investigating the coexistence of diabetes, depression, and anxiety as a means to prevent the onset of other diseases, such as Alzheimer’s, among Mexican-origin adults living in the US–Mexico border.

The present study sought to provide a more current surveillance estimate of diabetes in Mexican-origin adults and to examine risk and protective factors in a community healthcare-receiving study population. Controlling for sociodemographic characteristics (i.e., sex, age, education, time living in the US, and county), this study aimed to examine whether hypertension, depression, and anxiety were statistically and significantly associated with diabetes among a cohort of Mexican-origin adults from a community sample within three border counties in Southern Arizona. It hypothesized that hypertension, depression, anxiety, and age were positively associated with diabetes among Mexican-origin adults in Southern Arizona. Conversely, it hypothesized that higher educational attainment and longer duration of residency in the US were negatively related to diabetes within this population. This investigation is relevant considering the documented low knowledge about prevention, incidence of glycosylated hemoglobin evaluation, and attendance at diabetic educational programs among this population in the US–Mexico border [25,26], a region characterized by high rates of poverty, under-education, and comorbid chronic conditions, as well as low levels of health insurance coverage and healthcare professionals [27]. This study may inform the scope of need and the refinement of culturally tailored programs for patients with diabetes in the Southern Arizona border.

## 2. Materials and Methods

### 2.1. Study and Intervention

This non-randomized study was undertaken as part of the Linking Individuals Needs to Community and Clinical Services (LINKS) project, a prospective-matched observational study [28]. The primary objective of LINKS was to establish clinical–community linkages between a community health center and a community-based partner in Yuma, Pima, and Santa Cruz, Arizona [28]. This intervention involved the active involvement of community health workers (CHWs) operating within clinical settings, who engaged in various recruitment methods (e.g., word of mouth, clinic waiting rooms, health fairs, and community settings) to identify Mexican-origin patients at risk of chronic diseases. Subsequently, these CHWs connected the identified patients with CHWs operating in community settings, utilizing channels, such as phone communication, REDCap, or in-person interactions. The CHWs then facilitated referrals to relevant community programs and services, including but not limited to chronic disease self-management programs, diabetes empowerment education programs, health rhythms, injury prevention initiatives, parks and recreation facilities, support groups, and walking groups. Additionally, they provided instruction on emotional well-being techniques [28]. By fostering collaborative efforts to address the specific needs of the participants, the CHWs established a community–clinical linkage model aimed at expanding the continuum of care from clinical settings to the wider community.

### 2.2. Sample and Setting

The study sample consisted of 213 Mexican-origin adults who completed baseline assessments. Inclusion criteria were as follows: age ≥21, being an English or a Spanish speaker and being at risk of or experiencing a chronic disease (according to individuals’ electronic health records on weight, height, body mass index, glycosylated hemoglobin, blood pressure, and blood lipids profile) [28]. Participants met with the CHWs in one of three federally qualified health centers (i.e., Pima, Yuma, or Santa Cruz County) to complete the baseline survey either in English or Spanish between July 2017 and September 2018. CHWs obtained written consent from all LINKS participants. The University of Arizona Institutional Review Board approved all stages of the research (i.e., 1612044741R001).

### 2.3. Questionnaire

The LINKS project was part of a community-based participatory study developed between an academic institution and a diverse group of community partners that included federally qualified health centers within each county (the only or major center there), health departments, and other community advocates. These partners collaborated in a synergistic manner throughout the entirety of the study, encompassing various stages, such as the conception and formulation of the Emotional Well-being Questionnaire [28]. The dependent variable (i.e., type 1 and 2 diabetes) and the independent variables (i.e., hypertension, depression, and anxiety) were measured using the following question: Do you have any of the following health problems—diabetes, hypertension, depression, and anxiety? Response categories scored 0 = no and 1 = yes. The control variables (i.e., sociodemographics) were measured as follows: Sex was a binary variable with values 0 = men and 1 = female; age was a continuous variable grouped in three categories—0 = 18–44, 1 = 45–64, 2 = ≥65; education was a continuous variable grouped as 0 = <12 years and 1 = ≥12 years; county was a nominal variable with values 0 = Pima, 1 = Yuma, and 2 = Santa Cruz; and time living in the US was a continuous variable grouped in three categories—0 = born in the US, 1 = born in Mexico and living in the US for ≤30 years, and 2 = born in Mexico and living in the US for >30 years (the cutoff point for these variables aimed to distribute the sample more equitably).

### 2.4. Data Analysis

In this study, missing data (<5% for all variables) were imputed using a predictive mean matching. This involved randomly filling missing values with observed donor values using the Mice package in R 4.1. Five imputations were generated, which were then combined with the original observed data. Descriptive statistics were employed to compare Mexican-origin individuals with and without self-reported diabetes, considering self-reported diseases and demographic characteristics. The Chi-square test and Mann–Whitney U test were used, and statistical significance was determined at α = 0.05. To explore the determinants of self-reported diabetes while adjusting for sociodemographic characteristics, both bivariable and multivariable logistic regression models were conducted. Interaction terms were examined to identify potential moderator variables, with inclusion in the final model if they were statistically significant at a *p*-value < 0.05. The data analysis was performed using R software, specifically version R 4.1.

## 3. Results

Table 1 presents a comprehensive overview of the study population, revealing that the majority of individuals were female (85.9%) and belonged to the age group of 45 to 64 years (49.3%). Moreover, more than half of the participants had an educational attainment of less than 12 years (54%) and were born in Mexico, having resided in the US for less than 30 years (46%). In comparison to the non-diabetes group, the diabetes group exhibited noteworthy distinctions. Specifically, a significantly higher proportion of individuals in the diabetes group had hypertension (60.7% vs. 28.7%, *p* < 0.001), and there was also a significantly higher proportion of individuals aged 65 or older (45.2% vs. 24%, *p* = 0.012). Conversely, when compared to the non-diabetes group, the diabetes group displayed contrasting characteristics. A lower proportion of individuals in the diabetes group were female (77.4% vs. 91.5%, *p* = 0.015), had an educational attainment of less than 12 years (45.7% vs. 66.7%, *p* = 0.006), and were born in Mexico but had resided in the US for less than 30 years (29.8% vs. 56.6%, *p* = 0.007). Lastly, no significant direct differences were observed between the diabetes and non-diabetes groups in terms of anxiety, depression, and location.

Table 2 displays two logistic regression models that incorporate imputed data: one without an interaction effect (Model 1) and one with an interaction effect (Model 2). In Model 1, it is assumed that the impact of the depression variable remains constant across the different categories of time living in the US. In other words, if there is a statistically significant association between depression and diabetes, it holds true regardless of whether respondents have resided in the US for more than 30 years or not. However, Model 2 reveals that this assumption is not valid. There exists a statistically significant interaction between depression and time living in the US. After adjusting for all covariates, the final model (Model 2) demonstrates that individuals with hypertension were 2.36 times more likely to have diabetes (95% CI: 1.15, 4.83) when compared to those without hypertension. Holding covariates at a fixed value, individuals with 12 or more years of educational attainment had the odds of having diabetes that were 0.29 times (95% CI: 0.14, 0.61) lower than the corresponding odds for individuals with less than 12 years of education. Regarding individuals with depression, the odds of having diabetes for those born in Mexico and living in the US for less than 30 years were 0.04 times (95% CI: 0, 0.42) lower than the odds for individuals without depression who were born in the US. The Mann–Whitney U test did not indicate a direct statistically significant difference between depression and time living in the US, underscoring the importance of incorporating the combined significant effect of depression and time living in the US on diabetes within the model. Anxiety, sex, age, education, and location did not exhibit significant main effects or interaction effects on depressive symptoms at a *p*-value < 0.05.

## 4. Discussion

This study utilized cross-sectional data from the LINKS project to examine the factors associated with diabetes among Mexican-origin adults who were at risk for chronic diseases in Pima, Yuma, and Santa Cruz, Arizona. The overall prevalence of diabetes in the LINKS cohort was found to be 39.4%, which is higher compared to previous reports on diabetes prevalence among other cohorts of Mexican-origin adults along the US–Mexico border, ranging from 7.4% to 25.3% [4,25,26,29]. However, this elevated prevalence was expected as the LINKS cohort specifically targeted individuals at risk for chronic diseases. The statistical analysis unveiled positive associations between diabetes and hypertension, as well as having an educational attainment of less than 12 years. Furthermore, the impact of depression on diabetes was influenced by individuals’ country of birth and their duration of residence in the US. Specifically, the negative effect of depression on diabetes was less prominent among those who were born in Mexico and had resided in the US for less than 30 years. It is important to note that, in terms of effect, magnitude, or strength, all associations except one (hypertension on diabetes) were weak. These findings underscore the ongoing need for integrated prevention and treatment healthcare services that specifically address the challenges associated with diabetes. The higher prevalence observed in the LINKS cohort highlights the importance of targeted interventions for at-risk populations, while recognizing the complexities introduced by factors, such as hypertension, educational attainment, and the interplay between depression and migration-related factors. By addressing these multifaceted aspects, healthcare services can better cater to the needs of Mexican-origin adults and effectively mitigate the impact of diabetes.

The positive relationship between hypertension and diabetes is consistent with previous studies among Mexican-origin adults [7,12,30]. Special attention requires increasing awareness and treatment for hypertension considering that the Latine in general are more likely than non-Latine whites to be aware and treated for diabetes but less likely to be aware and treated for hypertension [30]. Specifically, it is particularly relevant to increase awareness of hypertension among Mexican-origin individuals considering that their treatment rates have been lower than those of non-Latine whites [30]. Additionally, diabetes and hypertension are an important focus for ischemic stroke screening and prevention among Mexican-origin individuals. The greater the commitment to treat diabetes and hypertension among Mexican-origin patients, the lower the risk of ischemic stroke [12].

Education and socioeconomic status are crucial social determinants that significantly influence the prevalence of diabetes in the US–Mexico border region [31,32,33]. These interconnected factors create barriers to accessing healthcare services, impeding individuals from improving their health, well-being, and overall quality of life [31,34]. Extensive research consistently indicates that lower education and socioeconomic status are associated with difficulties in diabetes management and an increased risk of diabetes-related complications [35,36]. Specifically, individuals with lower socioeconomic status face higher risks of encountering challenges in diabetes management and experiencing both short- and long-term complications, such as retinopathy and cardiopathy [35]. Conversely, access to education has been linked to improvements in clinical outcomes (glycemia, cholesterol), self-management practices (glucose self-monitoring, exercise, and diet), knowledge, and psychosocial well-being related to diabetes [36]. This study further supports this notion by demonstrating that individuals with higher educational attainment were less likely to have diabetes compared to their counterparts. That is, a low level of educational attainment affects health literacy and English proficiency in Mexican-origin people with diabetes, leading to distress and poorer diabetes self-care [37]. In addition to the impact of education and income, Mexican-origin individuals have the lowest rates of healthcare utilization among different groups in the US, primarily due to barriers that hinder access to care [38]. Therefore, promoting CHWs interventions is crucial as they facilitate education and communication regarding healthcare, enabling patients to better understand the healthcare system and receive the necessary support and services they require [31]. By implementing CHWs interventions, patients can bridge gaps in health literacy, improve their understanding of healthcare processes, and receive the support necessary to navigate the complex healthcare system effectively.

Approximately 32% and 24% of Mexican-origin adults who reported having diabetes also had depression and anxiety, respectively, which is consistent with prior research [39,40]. The existing literature lacks a consensus regarding the significant association between diabetes and depression when controlling for comorbid diseases [18]. Therefore, future studies conducted among Mexican-origin individuals with diabetes and depression in the US–Mexico border region should take into consideration the presence of comorbid diseases and adjust their analyses accordingly. Additionally, it is not clear whether diabetes causes depression and anxiety, or vice versa, or whether these diseases have a common underlying etiology mechanism or not [41]. The psychological stress associated with diabetes management (e.g., dietary changes, medication adherence, physical activity, and monitoring blood glucose levels) may lead to depressive and anxiety symptoms [19,42,43]. In general, depression and anxiety are often not diagnosed or underdiagnosed among Latine adults with diabetes due to cultural and linguistic barriers between health professionals and patients [24,39]. Thus, emotional well-being is critical to diabetes control [38,44], and these findings point toward the need to screen for depression and anxiety specifically among Mexican-origin individuals with diabetes during regular office visits [19].

These findings also points toward the need for consideration of the integration of primary care and mental health services to improve patients’ outcomes linked to diabetes [45]. Among low-income Latine with diabetes from five safety-net clinics in Los Angeles, California (n = 964), those receiving supportive care for the comorbid condition of depression and diabetes (n = 484) significantly decreased depression scores in a six month period (least squares mean (LSM) = 6.34, SE = 0.49) compared to those who received usual care (LSM = 5.08, SE = 0.48) [20]. Likewise, among low-income Latine with chronic physical and mental health conditions in a 12-county region along the Texan US–Mexico border, those who received integrated care services (n = 2254) had an average lower score for depression (0.39 points lower at 12 months) and for HbA1c (0.14 percentage points lower at 12 months) than those who received standard care (n = 1972) [45]. Programs training Mexican-origin adults on how to manage their diabetes and associated mental health issues (e.g., SONRISA in Arizona) could mitigate the negative repercussions of a growing population with diabetes and comorbid disability [24].

Past traumatic experiences (e.g., interpersonal, physical, or sexual abuse), and current social isolation and stress (e.g., health, family, financial, immigration, or workplace stress) are important determinants of depression among Mexican-origin individuals with diabetes [22,44]. However, this study found that individuals who were born in Mexico and who were living in the US for less than 30 years were less likely to have diabetes than their counterparts. It is not clear whether acculturation, the exposure and adaptation to a new culture, is related to higher prevalence of diabetes among Mexican-origin adults [4]. Thus, future studies should consider how acculturation may contribute to depression among Mexican-origin individuals with diabetes in southern Arizona [44].

This study observed that, at a marginally significant level (*p* < 0.1), older adults exhibited better glycemic control, potentially due to a lower degree of insulin resistance and higher participation/engagement in programs [2]. Furthermore, being female showed a marginal association with diabetes. However, previous research among Mexican-origin individuals residing in the US–Mexico border has not provided conclusive evidence regarding the relationship between sex and diabetes [2,4,25,26,29]. Notably, one study found that females had higher levels of physical activity, while males were more likely to adhere to a daily healthy eating plan; both physical activity and a daily healthful eating plan are strongly related to diabetes prevention and management [46]. Consequently, additional research is warranted to investigate the association between sex and diabetes. In conclusion, while there was a marginal association between being older/female and diabetes, the relationship between age/sex and diabetes among Mexican-origin individuals in the US–Mexico border region remains inconclusive. Future research should delve deeper into understanding the nuanced interplay between age/sex, lifestyle factors, and diabetes to inform targeted prevention and management strategies.

### Limitations

The cross-sectional data obtained from the LINKS study demonstrates both strengths and limitations. Noteworthy strengths include its focus on a high-need population residing in an underserved area. Additionally, the study implements a CHWs model, in which CHWs play a crucial role in facilitating connections between participants and various health and social services. Furthermore, the research benefits from extensive and continuous engagement with community organizations, ensuring valuable insights to inform all aspects of the study. However, there are several limitations worth noting. One significant limitation of the LINKS questionnaire is its exclusive reliance on self-reports for diagnosing diabetes, lacking the inclusion of laboratory investigations and physician diagnosis, which provide objective measures, such as glycosylated hemoglobin. To address this limitation, future research should aim to compare and incorporate both objective and subjective indicators of diabetes. Likewise, it did not distinguish between type 1 and type 2 diabetes [2], neither did it contain an evaluation of this and other variables through time (e.g., hypertension). Furthermore, it did not include measurements of healthy lifestyles (e.g., nutrition and exercise) or community–social determinants of health (e.g., income, occupation, health insurance, family history, among others), which may serve as potential risk factors for diabetes development and management [2,47]. Future studies focusing on diabetes among Mexican-origin adults in the US–Mexico border should consider including these measurements. Likewise, given that only a single item was used to assess depression and anxiety in this analysis, it did not allow for a distinction between disorders and symptoms [48]. It is recommended that future studies utilize separate instruments to evaluate both disorders and symptoms of both variables to gain a more comprehensive understanding of the subject. Additionally, the findings of this study are based on a convenient sample of Mexican-origin adults, predominantly female, who self-identified as being at risk for chronic diseases and who attend community health clinics in Yuma, Pima, and Santa Cruz, Arizona. Thus, future research could benefit from comparing a sample that is equally distributed in terms of sex and age between those who attend community health clinics and those who do not along the US–Mexico border. Given the specific sample and cross-sectional nature of this study, establishing generalizability of findings or temporal relationships were not possible in the analysis conducted. Finally, Model 2 revealed marginally significant effects of anxiety, sex, and age on depression. Therefore, future research should explore these associations in more depth, such as through longitudinal analyses.

## 5. Conclusions

This study contributes to a growing body of literature indicating an association between hypertension, depression, anxiety, and diabetes [42]. Future studies should determine whether interventions aimed at modifying behavioral factors associated with hypertension, depression, and anxiety will complement current diabetes prevention and control strategies. These findings suggest that clinicians should be aware of the increasing risk of elevated hypertension and depressive symptoms in adults with diabetes and consider routine screening for such symptoms among these patients; targeting screening to the highest-risk individuals is important. Diabetes prevention programs that are culturally adapted may be more effective in reducing the risk of diabetes among Mexican-origin adults [26]; interventions on diabetes management focusing on the social determinants of health are scarce but also necessary [2].

## Figures and Tables

**Table 1:** Baseline characteristics of the LINKS sample by self-report of diabetes.

	No (N = 129)	Yes (N = 84)	Total (N = 213)	*p*-Value
Hypertension				
No	92 (71.3%)	33 (39.3%)	125 (58.7%)	<0.001
Yes	37 (28.7%)	51 (60.7%)	88 (41.3%)	
Anxiety				
No	91 (70.5%)	64 (76.2%)	155 (72.8%)	0.664
Yes	38 (29.5%)	20 (23.8%)	58 (27.2%)	
Depression				
No	99 (76.7%)	57 (67.9%)	156 (73.2%)	0.359
Yes	30 (23.3%)	27 (32.1%)	57 (26.8%)	
Sex				
Male	11 (8.5%)	19 (22.6%)	30 (14.1%)	0.015
Female	118 (91.5%)	65 (77.4%)	183 (85.9%)	
Age (years)				
18–44	30 (23.3%)	8 (9.5%)	38 (17.8%)	0.012
45–64	67 (51.9%)	38 (45.2%)	105 (49.3%)	
>=65	31 (24.0%)	38 (45.2%)	69 (32.4%)	
Missing	1 (0.8%)	0 (0%)	1 (0.5%)	
Education (years)				
<12	59 (45.7%)	56 (66.7%)	115 (54.0%)	0.006
>=12	66 (51.2%)	24 (28.6%)	90 (42.3%)	
Missing	4 (3.1%)	4 (4.8%)	8 (3.8%)	
Place of Birth and Time in US (years)				
US Birth	18 (14.0%)	18 (21.4%)	36 (16.9%)	0.007
MX Birth and US <=30	73 (56.6%)	25 (29.8%)	98 (46.0%)	
MX Birth and US >30	35 (27.1%)	37 (44.0%)	72 (33.8%)	
Missing	3 (2.3%)	4 (4.8%)	7 (3.3%)	
County				
Pima	66 (51.2%)	27 (32.1%)	93 (43.7%)	0.084
Yuma	39 (30.2%)	31 (36.9%)	70 (32.9%)	
Santa Cruz	24 (18.6%)	26 (31.0%)	50 (23.5%)	

US = United States and MX = Mexico.

**Table 2:** Multivariable logistic regression models of the determinants of diabetes among Mexican-origin adults in Pima, Yuma, and Santa Cruz, Arizona.

	Model 1	Model 2
Hypertension (Yes)	2.61 **	2.36 *
	[1.30, 5.26]	[1.15, 4.83]
Anxiety (Yes)	0.41 +	0.39 +
	[0.17, 1.02]	[0.15, 1.01]
Depression (Yes)	1.52	5.58 +
	[0.65, 3.58]	[0.98, 31.94]
Sex (Female)	0.45 +	0.43 +
	[0.18, 1.15]	[0.16, 1.12]
Age (45-64 years)	2.18	2.27
	[0.72, 6.63]	[0.76, 6.81]
Age (≥65 years)	2.25	2.82 +
	[0.69, 7.35]	[0.85, 9.37]
Education (≥12 years)	0.32 **	0.29 **
	[0.16, 0.68]	[0.14, 0.61]
MX Birth and US ≤30 years	0.22 **	0.43
	[0.08, 0.60]	[0.14, 1.37]
MX Birth and US >30 years	0.35 *	0.39
	[0.13, 0.97]	[0.12, 1.31]
County (Yuma)	1.37	1.61
	[0.62, 3.02]	[0.71, 3.66]
County (Santa Cruz)	1.88	1.79
	[0.80, 4.46]	[0.73, 4.41]
Depression (Yes) * MX Birth and US ≤30 years		0.04 **
		[0.00, 0.42]
Depression (Yes) * MX Birth and US >30 years		0.57
		[0.08, 4.25]
N	213	213

US = United States and MX = Mexico. + *p* < 0.1, * *p* < 0.05, ** *p* < 0.01.

## Data Availability

The data presented in this study are available on request from the corresponding author.
